# 2-[2-(4-Acetyl­phen­yl)hydrazinyl­idene]-1,3-diphenyl­propane-1,3-dione

**DOI:** 10.1107/S1600536811027590

**Published:** 2011-07-16

**Authors:** Carlos Bustos, Daniela Barría, Luis Alvarez-Thon, Juan-Guillermo Cárcamo, Maria Teresa Garland

**Affiliations:** a Instituto de Ciencias Químicas, Universidad Austral de Chile, Avda. Los Robles s/n, Campus Isla Teja, Casilla 567, Valdivia, Chile; b Departamento de Ciencias Físicas, Universidad Andres Bello, Avda. República 220, Santiago de Chile, Chile; c Instituto de Ciencias Moleculares y Microbiología, Universidad Austral de Chile, Avda. Los Robles s/n, Campus Isla Teja, Casilla 567, Valdivia, Chile; d Laboratorio de Cristalografía, Departamento de Física, Facultad de Ciencias Físicas y Matemáticas, Universidad de Chile, Av. Blanco Encalada 2008, Santiago de Chile, Chile

## Abstract

In the title compound, C_23_H_18_N_2_O_3_, the inter­planar angle between the benzoyl units is 80.51 (6)° while the dihedral angles between the hydrazinyl­idene and benzoyl groups are 43.43 (6) and 54.16 (6)°. In the crystal, a strong resonance-assisted intra­molecular N—H⋯O hydrogen bond is observed. The mol­ecules form an inversion dimer *via* a pair of weak C—H⋯O hydrogen bonds and a π–π inter­action [centroid–centroid distance of 3.5719 (10) Å]. These dimers are linked *via* weak C—H⋯O contacts, forming chains along the *b* axis.

## Related literature

For details of the synthesis, see: Yao (1964 ▶). For resonance-assisted hydrogen bonds and related structures see: Bertolasi *et al.* (1993 ▶); Bustos, Alvarez-Thon, Barría, Cárcamo & Garland (2011 ▶); Bustos, Alvarez-Thon, Barría, Garland & Sánchez (2011 ▶); Bustos, Alvarez-Thon, Cárcamo, Garland & Sánchez (2011 ▶); Bustos, Alvarez-Thon, Cárcamo, Ibañez & Sánchez (2011 ▶); Gilli *et al.* (1993 ▶).
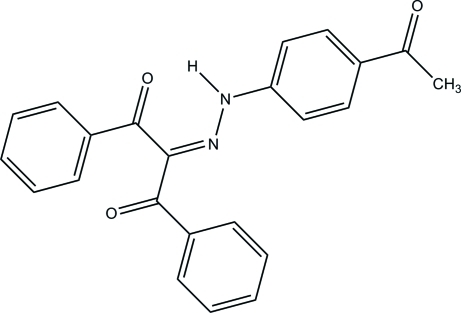


## Experimental

### 

#### Crystal data

C_23_H_18_N_2_O_3_
*M* *_r_* = 370.39
Monoclinic, 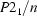

*a* = 12.6026 (15) Å
*b* = 11.0138 (13) Å
*c* = 14.9701 (18) Å
β = 114.447 (2)°
*V* = 1891.6 (4) Å^3^
*Z* = 4
Mo *K*α radiation
μ = 0.09 mm^−1^
*T* = 297 K
0.53 × 0.23 × 0.20 mm

#### Data collection

Bruker D8 Discover with SMART CCD area-detector diffractometer
14867 measured reflections
3869 independent reflections
2580 reflections with *I* > 2σ(*I*)
*R*_int_ = 0.047

#### Refinement

*R*[*F* ^2^ > 2σ(*F* ^2^)] = 0.042
*wR*(*F* ^2^) = 0.117
*S* = 0.97
3869 reflections
258 parameters
H atoms treated by a mixture of independent and constrained refinement
Δρ_max_ = 0.23 e Å^−3^
Δρ_min_ = −0.14 e Å^−3^

### 

Data collection: *SMART* (Bruker, 2001 ▶); cell refinement: *SAINT* (Bruker, 2000 ▶); data reduction: *SAINT*; program(s) used to solve structure: *SHELXS97* (Sheldrick, 2008 ▶); program(s) used to refine structure: *SHELXL97* (Sheldrick, 2008 ▶); molecular graphics: *XP* in *SHELXTL-PC *(Sheldrick, 2008 ▶); software used to prepare material for publication: *PLATON* (Spek, 2009 ▶) and *Mercury* (Macrae *et al.*, 2008 ▶).

## Supplementary Material

Crystal structure: contains datablock(s) global, I. DOI: 10.1107/S1600536811027590/fj2443sup1.cif

Structure factors: contains datablock(s) I. DOI: 10.1107/S1600536811027590/fj2443Isup2.hkl

Supplementary material file. DOI: 10.1107/S1600536811027590/fj2443Isup3.cml

Additional supplementary materials:  crystallographic information; 3D view; checkCIF report

## Figures and Tables

**Table 1:** Hydrogen-bond geometry (Å, °)

*D*—H⋯*A*	*D*—H	H⋯*A*	*D*⋯*A*	*D*—H⋯*A*
N2—H1⋯O2	0.888 (15)	1.965 (15)	2.6496 (16)	132.8 (15)
C15—H15⋯O3^i^	0.93	2.38	3.2503 (19)	156
C21—H21⋯O1^ii^	0.93	2.67	3.2983 (18)	125

Symmetry codes: (i) 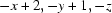; (ii) 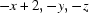.

**Fig. 1.**
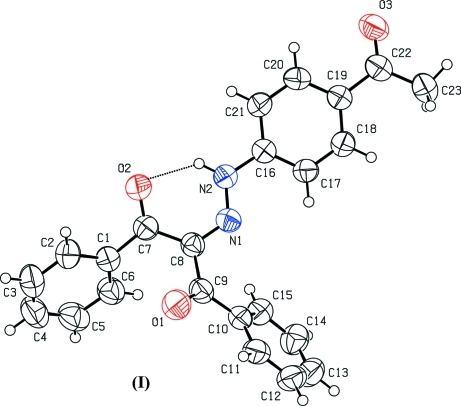
View of the title compound with the atom numbering scheme. Displacement ellipsoids for non-H atoms are drawn at the 50% probability level. The strong intramolecular hydrogen bond (N2–H1···O2) is depicted with a dashed line.

**Fig. 2.**
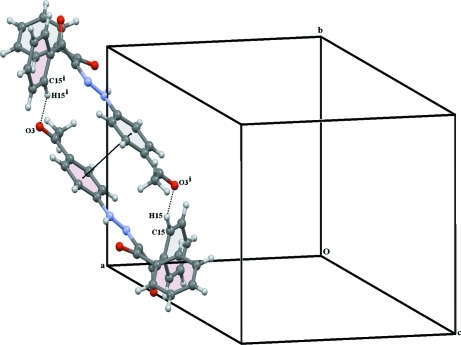
Part of the crystal packing showing the formation of an inversion-related dimer via C15—H15···O3i weak contacts (dashed lines) and a π-π stacking interaction (solid line) [symmetry code: (i) -x + 2, -y + 1, -z].

**Fig. 3.**
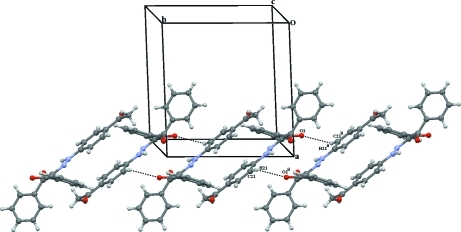
Part of the crystal packing showing the formation of a chain along the b axis, by linking dimers (see Fig. 2) through weak C21—H21···O1ii contacts (dashed lines) [symmetry code: (ii) -x + 2, -y, -z].

## supplementary crystallographic information

### Comment

β-diketones are known to form strong intramolecular O—H···O hydrogen bonds where the decrease of the O···O contact distance (up to 2.40 Å) is correlated with the increased π-delocalization of the O—C=C—C=O heteroconjugated system; the phenomenon has been interpreted by the Resonance Assisted Hydrogen Bond (RAHB) model (Gilli *et al.*, 1993). Besides, this concept has been applied to other heterodienic systems such as enaminones and ketohydrazones (Bertolasi *et al.*, 1993). On the other hand, in previous works we have reported the crystalline structures of three β-diketohydrazones of the type 2-(2-(*R*-phenyl)hydrazinylidene)-1,3-diphenylpropane-1,3-dione (*R* = 4-Br, 4-NO_2_, 3-Cl) (Bustos, Alvarez-Thon, Cárcamo, Garland and Sánchez, 2011; Bustos, Alvarez-Thon, Cárcamo, Ibañez & Sánchez, 2011; Bustos, Alvarez-Thon, Barría, Garland & Sánchez, 2011) and a second polymorph of 2-(2-(4-methoxyphenyl)hydrazinylidene)-1,3-diphenylpropane-1,3-dione (Bustos, Alvarez-Thon, Barría, Cárcamo & Garland, 2011), containing this hydrogen-bonded core. Now, we present the title compound prepared using similar methodology (Yao, 1964).

The molecular structure of the title compound, (I), is shown in Fig. 1. In (I), the interplanar angle between the benzoyl units is 80.51 (6)°. The corresponding angles between the hydrazinylidene and the benzoyl groups are 43.43 (6) and 54.16 (6)°, respectively. In (I), a strong resonance-assisted intramolecular hydrogen bond N2—H1···O2 is observed (Fig. 1, Table 1). In the crystal, the entire supramolecular structure is constructed by weak intermolecular interactions. The molecules form an inversion dimer *via* a pair of weak C15—H15···O3^i^ hydrogen bonds and an inter-ring π-π interaction with a centroid-centroid distance of 3.5719 (10) Å, (Fig. 2, Table1), and these dimers are linked *via* weak C21—H21···O1^ii^ contacts to form chains along the *b* axis (Table 1, Fig. 3) [symmetry codes: (i) -*x* + 2, -*y* + 1, -*z*; (ii) -*x* + 2, -*y*, -*z*].

### Experimental

Chemicals: 1,3-diphenylpropane-1,3-dione, 4-aminoacetophenone and sodium nitrite were procured from Sigma-Aldrich and sodium hydroxide, hydrochloric acid, sodium acetate and solvents from Merck. These chemicals were used without previous purification.

Procedure: In a 500 ml beaker flask were dissolved 2.29 g (0.01 mole) of 1,3-diphenylpropane-1,3-dione (98%) in 100 ml of an ethanol solution containing 0.4 g (0.01 mole) of sodium hydroxide. This solution was then buffered by adding 4.80 g of sodium acetate trihydrate. The resulting β-diketonate solution was diluted with water to a volume of about 220 ml, and stirred and cooled at -5 °C. On the other hand, in another 50 ml beaker flask, a diazonium ion solution was prepared adding 1.36 g (0.01 mole) of 4-aminoacetophenone (99%) in 8 ml of hydrochloric acid (5 mol/*L*), cooling at -5 °C, and adding dropwise a saturated aqueous solution containing 0.69 g (0.01 mole) of sodium nitrite. The diazonium salt solution was then added dropwise with vigorous stirring into the buffered β-diketonate solution. During the addition an orange solid was observed. This precipitate was filtered by suction and washed with an abundant quantity of water. Yield: 95% of crude product. Single crystals suitable for X-ray studies were obtained by recrystallization from a concentrated solution of the compound in ethanol.

### Refinement

All hydrogen atoms were found in difference Fourier maps. The hydrogen attached to N2 was refined freely against the diffraction data, but all other H atoms were placed in geometrically idealized positions and constrained to ride on their parent atoms, with aromatic C—H = 0.93 Å, methyl C—H = 0.96 Å and *U*_iso_(H) = 1.2*U*_eq_(aromatic C) or *U*_iso_(H) = 1.5*U*_eq_(aliphatic C).

### Crystal data

**Table:**

C_23_H_18_N_2_O_3_	*F*(000) = 776
*M**_r_* = 370.39	*D*_x_ = 1.301 Mg m^−^^3^
Monoclinic, *P*2_1_/*n*	Mo *K*α radiation, λ = 0.71073 Å
Hall symbol: -P 2yn	Cell parameters from 999 reflections
*a* = 12.6026 (15) Å	θ = 1.8–26.4°
*b* = 11.0138 (13) Å	µ = 0.09 mm^−^^1^
*c* = 14.9701 (18) Å	*T* = 297 K
β = 114.447 (2)°	Polyhedron, yellow
*V* = 1891.6 (4) Å^3^	0.53 × 0.23 × 0.20 mm
*Z* = 4	

### Data collection

**Table:**

Bruker D8 Discover with SMART CCD area-detector diffractometer	2580 reflections with *I* > 2σ(*I*)
Radiation source: fine-focus sealed tube	*R*_int_ = 0.047
graphite	θ_max_ = 26.4°, θ_min_ = 1.8°
φ and ω scans	*h* = −15→15
14867 measured reflections	*k* = −13→13
3869 independent reflections	*l* = −18→18

### Refinement

**Table:**

Refinement on *F*^2^	Primary atom site location: structure-invariant direct methods
Least-squares matrix: full	Secondary atom site location: difference Fourier map
*R*[*F*^2^ > 2σ(*F*^2^)] = 0.042	Hydrogen site location: inferred from neighbouring sites
*wR*(*F*^2^) = 0.117	H atoms treated by a mixture of independent and constrained refinement
*S* = 0.97	*w* = 1/[σ^2^(*F*_o_^2^) + (0.0642*P*)^2^] where *P* = (*F*_o_^2^ + 2*F*_c_^2^)/3
3869 reflections	(Δ/σ)_max_ < 0.001
258 parameters	Δρ_max_ = 0.23 e Å^−^^3^
0 restraints	Δρ_min_ = −0.14 e Å^−^^3^

### Special details

**Table:**

Geometry. Bond distances, angles *etc*. have been calculated using the rounded fractional coordinates. All su's are estimated from the variances of the (full) variance-covariance matrix. The cell e.s.d.'s are taken into account in the estimation of distances, angles and torsion angles
Refinement. Refinement on *F*^2^ for ALL reflections except those flagged by the user for potential systematic errors. Weighted *R*-factors *wR* and all goodnesses of fit *S* are based on *F*^2^, conventional *R*-factors *R* are based on *F*, with *F* set to zero for negative *F*^2^. The observed criterion of *F*^2^ > σ(*F*^2^) is used only for calculating -*R*-factor-obs *etc*. and is not relevant to the choice of reflections for refinement. *R*-factors based on *F*^2^ are statistically about twice as large as those based on *F*, and *R*-factors based on ALL data will be even larger.

### Fractional atomic coordinates and isotropic or equivalent isotropic displacement parameters (Å^2^)

**Table:**

	*x*	*y*	*z*	*U*_iso_*/*U*_eq_	
O1	0.86013 (11)	−0.08254 (9)	0.12281 (8)	0.0874 (5)	
O2	0.86709 (9)	0.03801 (9)	−0.11984 (7)	0.0738 (4)	
O3	1.32598 (10)	0.65485 (10)	0.01670 (8)	0.0856 (5)	
N1	0.94625 (10)	0.17881 (9)	0.05722 (8)	0.0550 (4)	
N2	0.98248 (10)	0.22190 (10)	−0.00751 (9)	0.0566 (4)	
C1	0.71117 (12)	−0.04300 (12)	−0.09060 (10)	0.0568 (5)	
C2	0.69701 (13)	−0.15056 (13)	−0.14206 (10)	0.0657 (5)	
C3	0.59340 (17)	−0.21286 (15)	−0.17400 (12)	0.0821 (6)	
C4	0.50186 (16)	−0.16661 (18)	−0.15818 (14)	0.0901 (7)	
C5	0.51404 (15)	−0.05861 (18)	−0.10926 (13)	0.0870 (7)	
C6	0.61877 (13)	0.00324 (14)	−0.07386 (11)	0.0712 (6)	
C7	0.82231 (12)	0.02578 (12)	−0.06118 (10)	0.0566 (5)	
C8	0.87475 (11)	0.08622 (12)	0.03603 (10)	0.0535 (4)	
C9	0.86337 (12)	0.02835 (13)	0.12137 (10)	0.0598 (5)	
C10	0.86295 (11)	0.10100 (12)	0.20482 (10)	0.0555 (5)	
C11	0.89533 (12)	0.04458 (14)	0.29535 (11)	0.0667 (5)	
C12	0.89283 (14)	0.10804 (18)	0.37354 (12)	0.0792 (7)	
C13	0.85639 (15)	0.22591 (18)	0.36290 (13)	0.0833 (7)	
C14	0.82327 (14)	0.28230 (16)	0.27368 (13)	0.0787 (6)	
C15	0.82714 (12)	0.22093 (13)	0.19482 (11)	0.0635 (5)	
C16	1.05800 (11)	0.32173 (11)	0.01612 (10)	0.0505 (4)	
C17	1.08787 (12)	0.38540 (12)	0.10315 (10)	0.0561 (5)	
C18	1.16533 (12)	0.48066 (12)	0.12415 (10)	0.0569 (5)	
C19	1.21201 (11)	0.51695 (11)	0.05924 (10)	0.0525 (4)	
C20	1.17882 (12)	0.45310 (13)	−0.02859 (10)	0.0589 (5)	
C21	1.10363 (12)	0.35601 (12)	−0.05004 (10)	0.0575 (5)	
C22	1.29307 (12)	0.62111 (13)	0.07860 (11)	0.0612 (5)	
C23	1.33418 (14)	0.68561 (13)	0.17546 (12)	0.0749 (6)	
H1	0.9663 (13)	0.1801 (13)	−0.0623 (11)	0.075 (5)*	
H2	0.75800	−0.18090	−0.15510	0.0790*	
H3	0.58550	−0.28660	−0.20640	0.0990*	
H4	0.43150	−0.20840	−0.18060	0.1080*	
H5	0.45110	−0.02670	−0.09980	0.1040*	
H6	0.62720	0.07520	−0.03910	0.0850*	
H11	0.91870	−0.03630	0.30310	0.0800*	
H12	0.91620	0.07020	0.43430	0.0950*	
H13	0.85400	0.26790	0.41590	0.1000*	
H14	0.79800	0.36250	0.26640	0.0940*	
H15	0.80570	0.26020	0.13480	0.0760*	
H17	1.05590	0.36400	0.14690	0.0670*	
H18	1.18690	0.52170	0.18340	0.0680*	
H20	1.20810	0.47660	−0.07360	0.0710*	
H21	1.08350	0.31360	−0.10850	0.0690*	
H23A	1.39270	0.74400	0.18000	0.1120*	
H23B	1.36650	0.62770	0.22770	0.1120*	
H23C	1.26960	0.72640	0.18060	0.1120*	

### Atomic displacement parameters (Å^2^)

**Table:**

	*U*^11^	*U*^22^	*U*^33^	*U*^12^	*U*^13^	*U*^23^
O1	0.1300 (10)	0.0525 (6)	0.0877 (8)	−0.0137 (6)	0.0532 (8)	−0.0034 (6)
O2	0.0706 (7)	0.0888 (8)	0.0710 (7)	−0.0153 (6)	0.0383 (6)	−0.0177 (5)
O3	0.0768 (8)	0.1003 (9)	0.0750 (7)	−0.0239 (6)	0.0268 (6)	0.0133 (6)
N1	0.0558 (7)	0.0521 (7)	0.0599 (7)	0.0007 (5)	0.0269 (6)	0.0023 (5)
N2	0.0609 (7)	0.0553 (7)	0.0565 (7)	−0.0054 (6)	0.0272 (6)	−0.0033 (6)
C1	0.0557 (8)	0.0551 (8)	0.0575 (8)	−0.0020 (6)	0.0213 (7)	−0.0008 (6)
C2	0.0651 (10)	0.0603 (9)	0.0624 (9)	0.0029 (7)	0.0171 (7)	−0.0030 (7)
C3	0.0845 (12)	0.0659 (10)	0.0779 (11)	−0.0140 (9)	0.0155 (9)	−0.0068 (8)
C4	0.0678 (12)	0.0955 (13)	0.0937 (13)	−0.0240 (10)	0.0202 (10)	−0.0023 (11)
C5	0.0634 (10)	0.1010 (13)	0.1007 (13)	−0.0049 (10)	0.0382 (10)	0.0007 (11)
C6	0.0660 (10)	0.0693 (9)	0.0839 (11)	−0.0041 (8)	0.0367 (9)	−0.0079 (8)
C7	0.0568 (8)	0.0532 (8)	0.0631 (9)	0.0026 (6)	0.0281 (7)	−0.0019 (6)
C8	0.0543 (8)	0.0482 (7)	0.0596 (8)	−0.0012 (6)	0.0251 (7)	−0.0032 (6)
C9	0.0611 (9)	0.0534 (9)	0.0659 (9)	−0.0073 (7)	0.0272 (7)	−0.0019 (7)
C10	0.0502 (8)	0.0592 (8)	0.0576 (8)	−0.0064 (6)	0.0229 (7)	−0.0008 (7)
C11	0.0641 (9)	0.0706 (9)	0.0678 (10)	−0.0048 (7)	0.0296 (8)	0.0065 (8)
C12	0.0730 (11)	0.1068 (14)	0.0602 (10)	−0.0058 (10)	0.0300 (9)	0.0045 (9)
C13	0.0783 (11)	0.1076 (14)	0.0682 (11)	−0.0035 (10)	0.0346 (9)	−0.0178 (10)
C14	0.0774 (11)	0.0805 (11)	0.0792 (11)	0.0091 (9)	0.0333 (9)	−0.0110 (9)
C15	0.0605 (9)	0.0684 (9)	0.0604 (9)	0.0038 (7)	0.0239 (7)	−0.0005 (7)
C16	0.0480 (7)	0.0482 (7)	0.0546 (8)	0.0045 (6)	0.0204 (6)	0.0029 (6)
C17	0.0609 (9)	0.0552 (8)	0.0573 (8)	0.0005 (7)	0.0295 (7)	0.0027 (6)
C18	0.0607 (9)	0.0543 (8)	0.0536 (8)	0.0006 (7)	0.0216 (7)	−0.0002 (6)
C19	0.0456 (7)	0.0527 (8)	0.0564 (8)	0.0050 (6)	0.0183 (6)	0.0061 (6)
C20	0.0549 (8)	0.0662 (9)	0.0621 (9)	0.0014 (7)	0.0306 (7)	0.0062 (7)
C21	0.0605 (9)	0.0604 (8)	0.0545 (8)	0.0001 (7)	0.0267 (7)	−0.0036 (7)
C22	0.0492 (8)	0.0612 (9)	0.0665 (9)	0.0032 (6)	0.0173 (7)	0.0137 (7)
C23	0.0701 (10)	0.0671 (9)	0.0803 (11)	−0.0129 (8)	0.0239 (9)	−0.0044 (8)

### Geometric parameters (Å, °)

**Table:**

O1—C9	1.2225 (17)	C16—C21	1.388 (2)
O2—C7	1.2321 (19)	C17—C18	1.378 (2)
O3—C22	1.219 (2)	C18—C19	1.387 (2)
N1—N2	1.3187 (18)	C19—C20	1.3939 (19)
N1—C8	1.3099 (18)	C19—C22	1.483 (2)
N2—C16	1.4004 (18)	C20—C21	1.376 (2)
N2—H1	0.888 (15)	C22—C23	1.501 (2)
C1—C2	1.384 (2)	C2—H2	0.9300
C1—C6	1.386 (2)	C3—H3	0.9300
C1—C7	1.490 (2)	C4—H4	0.9300
C2—C3	1.374 (3)	C5—H5	0.9300
C3—C4	1.368 (3)	C6—H6	0.9300
C4—C5	1.372 (3)	C11—H11	0.9300
C5—C6	1.381 (3)	C12—H12	0.9300
C7—C8	1.4838 (19)	C13—H13	0.9300
C8—C9	1.487 (2)	C14—H14	0.9300
C9—C10	1.485 (2)	C15—H15	0.9300
C10—C15	1.384 (2)	C17—H17	0.9300
C10—C11	1.390 (2)	C18—H18	0.9300
C11—C12	1.375 (2)	C20—H20	0.9300
C12—C13	1.364 (3)	C21—H21	0.9300
C13—C14	1.372 (3)	C23—H23A	0.9600
C14—C15	1.379 (2)	C23—H23B	0.9600
C16—C17	1.3874 (19)	C23—H23C	0.9600
			
O1···C1	2.9927 (18)	C8···H6	2.8500
O1···C6	3.377 (2)	C8···H15	2.7700
O1···C21^i^	3.2983 (18)	C9···H6	3.0000
O2···C8^i^	3.2639 (19)	C11···H14^vii^	2.9900
O2···N1	2.8700 (15)	C12···H20^viii^	2.9100
O2···N2	2.6496 (16)	C14···H4^vi^	3.0300
O2···N1^i^	3.2078 (16)	C16···H2^i^	2.8500
O3···C15^ii^	3.2503 (19)	C17···H2^i^	2.8600
O3···C12^iii^	3.272 (2)	C18···H23C	2.9700
O1···H11	2.5400	C18···H23B	2.8600
O1···H21^i^	2.6700	C23···H18	2.6300
O1···H1^i^	2.898 (17)	C23···H2^ix^	3.0600
O2···H2	2.7200	H1···O2	1.965 (15)
O2···H23B^iv^	2.9200	H1···C7	2.491 (16)
O2···H1	1.965 (15)	H1···H21	2.3800
O3···H20	2.5000	H1···O1^i^	2.898 (17)
O3···H15^ii^	2.3800	H2···O2	2.7200
N1···O2	2.8700 (15)	H2···C16^i^	2.8500
N1···C15	3.045 (2)	H2···C17^i^	2.8600
N1···O2^i^	3.2078 (16)	H2···C23^iv^	3.0600
N2···O2	2.6496 (16)	H2···H23C^iv^	2.5700
N1···H17	2.5200	H4···C14^vi^	3.0300
N1···H15	2.6400	H5···C6^vi^	3.0800
C1···O1	2.9927 (18)	H6···C8	2.8500
C2···C16^i^	3.439 (2)	H6···C9	3.0000
C6···O1	3.377 (2)	H11···O1	2.5400
C6···C9	3.266 (2)	H14···C11^x^	2.9900
C8···O2^i^	3.2639 (19)	H15···N1	2.6400
C9···C6	3.266 (2)	H15···C8	2.7700
C12···O3^v^	3.272 (2)	H15···O3^ii^	2.3800
C15···O3^ii^	3.2503 (19)	H17···N1	2.5200
C15···N1	3.045 (2)	H18···C23	2.6300
C16···C19^ii^	3.582 (2)	H18···H23B	2.3800
C16···C2^i^	3.439 (2)	H18···H23C	2.4900
C16···C18^ii^	3.489 (2)	H18···C2^ix^	2.9300
C17···C20^ii^	3.550 (2)	H20···O3	2.5000
C18···C16^ii^	3.489 (2)	H20···C12^xi^	2.9100
C18···C21^ii^	3.584 (2)	H21···H1	2.3800
C19···C16^ii^	3.582 (2)	H21···O1^i^	2.6700
C20···C17^ii^	3.550 (2)	H23B···C18	2.8600
C21···O1^i^	3.2983 (18)	H23B···H18	2.3800
C21···C18^ii^	3.584 (2)	H23B···O2^ix^	2.9200
C2···H18^iv^	2.9300	H23C···C18	2.9700
C6···H5^vi^	3.0800	H23C···H18	2.4900
C7···H1	2.491 (16)	H23C···H2^ix^	2.5700
			
N2—N1—C8	120.99 (11)	C16—C21—C20	119.59 (13)
N1—N2—C16	120.18 (11)	C19—C22—C23	119.30 (13)
N1—N2—H1	118.4 (10)	O3—C22—C19	120.71 (13)
C16—N2—H1	120.9 (11)	O3—C22—C23	119.99 (14)
C6—C1—C7	121.16 (13)	C1—C2—H2	120.00
C2—C1—C6	119.40 (15)	C3—C2—H2	120.00
C2—C1—C7	119.26 (14)	C2—C3—H3	120.00
C1—C2—C3	120.42 (16)	C4—C3—H3	120.00
C2—C3—C4	120.11 (16)	C3—C4—H4	120.00
C3—C4—C5	119.94 (19)	C5—C4—H4	120.00
C4—C5—C6	120.70 (19)	C4—C5—H5	120.00
C1—C6—C5	119.37 (15)	C6—C5—H5	120.00
C1—C7—C8	120.05 (13)	C1—C6—H6	120.00
O2—C7—C1	119.79 (12)	C5—C6—H6	120.00
O2—C7—C8	120.03 (13)	C10—C11—H11	120.00
N1—C8—C9	115.27 (12)	C12—C11—H11	120.00
N1—C8—C7	124.70 (13)	C11—C12—H12	120.00
C7—C8—C9	119.22 (12)	C13—C12—H12	120.00
O1—C9—C10	120.77 (13)	C12—C13—H13	120.00
O1—C9—C8	117.34 (13)	C14—C13—H13	120.00
C8—C9—C10	121.80 (12)	C13—C14—H14	120.00
C9—C10—C15	122.53 (13)	C15—C14—H14	120.00
C11—C10—C15	118.90 (13)	C10—C15—H15	120.00
C9—C10—C11	118.52 (12)	C14—C15—H15	120.00
C10—C11—C12	120.13 (15)	C16—C17—H17	120.00
C11—C12—C13	120.64 (16)	C18—C17—H17	120.00
C12—C13—C14	119.77 (17)	C17—C18—H18	119.00
C13—C14—C15	120.49 (16)	C19—C18—H18	119.00
C10—C15—C14	120.06 (14)	C19—C20—H20	119.00
N2—C16—C21	118.19 (12)	C21—C20—H20	119.00
C17—C16—C21	120.08 (13)	C16—C21—H21	120.00
N2—C16—C17	121.73 (13)	C20—C21—H21	120.00
C16—C17—C18	119.37 (14)	C22—C23—H23A	109.00
C17—C18—C19	121.70 (13)	C22—C23—H23B	109.00
C18—C19—C20	117.83 (13)	C22—C23—H23C	109.00
C20—C19—C22	119.22 (13)	H23A—C23—H23B	109.00
C18—C19—C22	122.94 (12)	H23A—C23—H23C	109.00
C19—C20—C21	121.41 (14)	H23B—C23—H23C	109.00
			
C8—N1—N2—C16	179.36 (13)	O1—C9—C10—C11	20.0 (2)
N2—N1—C8—C7	−3.8 (2)	C8—C9—C10—C11	−156.37 (15)
N2—N1—C8—C9	165.87 (13)	C8—C9—C10—C15	26.3 (2)
N1—N2—C16—C17	−6.4 (2)	C9—C10—C11—C12	−178.17 (16)
N1—N2—C16—C21	173.30 (13)	C11—C10—C15—C14	−0.4 (2)
C6—C1—C2—C3	−1.8 (2)	C15—C10—C11—C12	−0.8 (2)
C2—C1—C7—C8	−141.11 (14)	C9—C10—C15—C14	176.90 (16)
C6—C1—C7—O2	−132.20 (15)	C10—C11—C12—C13	1.4 (3)
C6—C1—C7—C8	43.78 (19)	C11—C12—C13—C14	−0.8 (3)
C7—C1—C2—C3	−176.99 (13)	C12—C13—C14—C15	−0.4 (3)
C2—C1—C6—C5	−0.3 (2)	C13—C14—C15—C10	1.0 (3)
C7—C1—C6—C5	174.80 (14)	N2—C16—C17—C18	178.04 (13)
C2—C1—C7—O2	42.92 (19)	C21—C16—C17—C18	−1.6 (2)
C1—C2—C3—C4	2.4 (2)	N2—C16—C21—C20	−179.48 (13)
C2—C3—C4—C5	−0.8 (3)	C17—C16—C21—C20	0.2 (2)
C3—C4—C5—C6	−1.3 (3)	C16—C17—C18—C19	1.8 (2)
C4—C5—C6—C1	1.8 (3)	C17—C18—C19—C20	−0.5 (2)
C1—C7—C8—C9	34.6 (2)	C17—C18—C19—C22	178.33 (14)
C1—C7—C8—N1	−156.14 (14)	C18—C19—C20—C21	−1.0 (2)
O2—C7—C8—N1	19.8 (2)	C22—C19—C20—C21	−179.85 (14)
O2—C7—C8—C9	−149.42 (14)	C18—C19—C22—O3	−175.13 (15)
N1—C8—C9—O1	−138.55 (15)	C18—C19—C22—C23	4.6 (2)
C7—C8—C9—C10	−151.87 (14)	C20—C19—C22—O3	3.7 (2)
C7—C8—C9—O1	31.7 (2)	C20—C19—C22—C23	−176.60 (14)
N1—C8—C9—C10	37.9 (2)	C19—C20—C21—C16	1.1 (2)
O1—C9—C10—C15	−157.37 (16)		

Symmetry codes: (i) −*x*+2, −*y*, −*z*; (ii) −*x*+2, −*y*+1, −*z*; (iii) −*x*+5/2, *y*+1/2, −*z*+1/2; (iv) *x*−1/2, −*y*+1/2, *z*−1/2; (v) −*x*+5/2, *y*−1/2, −*z*+1/2; (vi) −*x*+1, −*y*, −*z*; (vii) −*x*+3/2, *y*−1/2, −*z*+1/2; (viii) *x*−1/2, −*y*+1/2, *z*+1/2; (ix) *x*+1/2, −*y*+1/2, *z*+1/2; (x) −*x*+3/2, *y*+1/2, −*z*+1/2; (xi) *x*+1/2, −*y*+1/2, *z*−1/2.

### Hydrogen-bond geometry (Å, °)

**Table:**

*D*—H···*A*	*D*—H	H···*A*	*D*···*A*	*D*—H···*A*
N2—H1···O2	0.888 (15)	1.965 (15)	2.6496 (16)	132.8 (15)
C15—H15···O3^ii^	0.93	2.38	3.2503 (19)	156
C21—H21···O1^i^	0.93	2.67	3.2983 (18)	125

Symmetry codes: (ii) −*x*+2, −*y*+1, −*z*; (i) −*x*+2, −*y*, −*z*.
